# Knowledge, attitudes, and practices (KAP) of caregivers regarding PM_2.5_ exposure among children in Northern Thailand: a cross-sectional study on the knowledge–practice gap

**DOI:** 10.1016/j.joclim.2026.100734

**Published:** 2026-08-19

**Authors:** Suparat Phuanukoonnon, Pyae Linn Aung, Wissanupong Kliengchuay, Sarima Niampardit, Nuttapohn Kiangkoo, Rachaneekorn Mingkhwan, Paweena Aendo, Sawaeng Kawichai, Kotchakorn Khammoon, Tippawan Prapamontol, Nguyen Thi Kim Oanh, Walaiporn Phonphan, Bin Jalaludin, Sotiris Vardoulakis, Kraichat Tantrakarnapa

**Affiliations:** a Department of Social and Environmental Medicine, Faculty of Tropical Medicine, Mahidol University, Bangkok, 10400, Thailand; b Mahidol Vivax Research Unit, Faculty of Tropical Medicine, Mahidol University, Bangkok, 10400, Thailand; c Research Institute for Health Sciences, Chiang Mai University, Chiang Mai, 50200, Thailand; d Environmental Engineering and Management, Asian Institute of Technology, Pathumthani, 12120, Thailand; e Faculty of Science and Technology, Suan Sunandha Rajabhat University, Bangkok, 10300, Thailand; f School of Population Health, University of New South Wales, NSW, Australia; g HEAL Global Research Centre, Health Research Institute, University of Canberra, Canberra, ACT, Australia

**Keywords:** PM_2.5_, Caregiver perceptions, Knowledge, Attitude, Practices, Child health, Northern Thailand

## Abstract

**Introduction:**

Fine particulate matter (PM_2.5_) poses severe threats to early childhood development through neurotoxic, respiratory, and immunological pathways. Developing effective public health interventions requires a deep understanding of caregiver perceptions and protective behaviors. This cross-sectional study assessed caregivers’ knowledge, attitudes, and practices (KAP) regarding PM_2.5_ exposure among children in Chiang Mai, Thailand.

**Methods:**

Caregivers were recruited at Child Development Centers during child drop-off or pick-up, with interviews scheduled at their homes or the centers. Structured questionnaires were administered to caregivers to evaluate KAP levels and identify socioeconomic predictors of protective behaviors.

**Results:**

Caregivers exhibited moderate knowledge (mean 10.44 ± 3.80 out of 22) and generally positive attitudes (30.74 ± 3.57 out of 40) toward PM_2.5_ protection. Although 97.0% recognized the health risks to children, critical technical gaps were identified: only 41.8% understood that standard surgical masks are insufficient for PM_2.5_, and only 9.7% could correctly interpret Air Quality Index (AQI) levels. Protective practices were inconsistent (17.33 ± 5.22 out of 32), characterized by low air purifier ownership (15.3%) and infrequent use of monitoring applications (28.2%). A significant socioeconomic gradient emerged, with higher education and income serving as robust predictors of preventive practices (adjusted R^2^ = 0.217). Correlations between knowledge, attitudes, and practices were weakly positive (r = 0.09 to 0.26), and 81.3% of caregivers reported feelings of hopelessness regarding pollution control.

**Conclusions:**

An apparent discrepancy between caregivers’ knowledge and protective practices was observed in Chiang Mai. Although high levels of concern are present, they do not always translate into effective protective actions, which may be influenced by technical misconceptions and socioeconomic barriers. Efforts focused on targeted education and subsidized protective measures could help mitigate the health burden of PM_2.5_ on vulnerable pediatric populations.

## Graphical abstract

**Figure:**
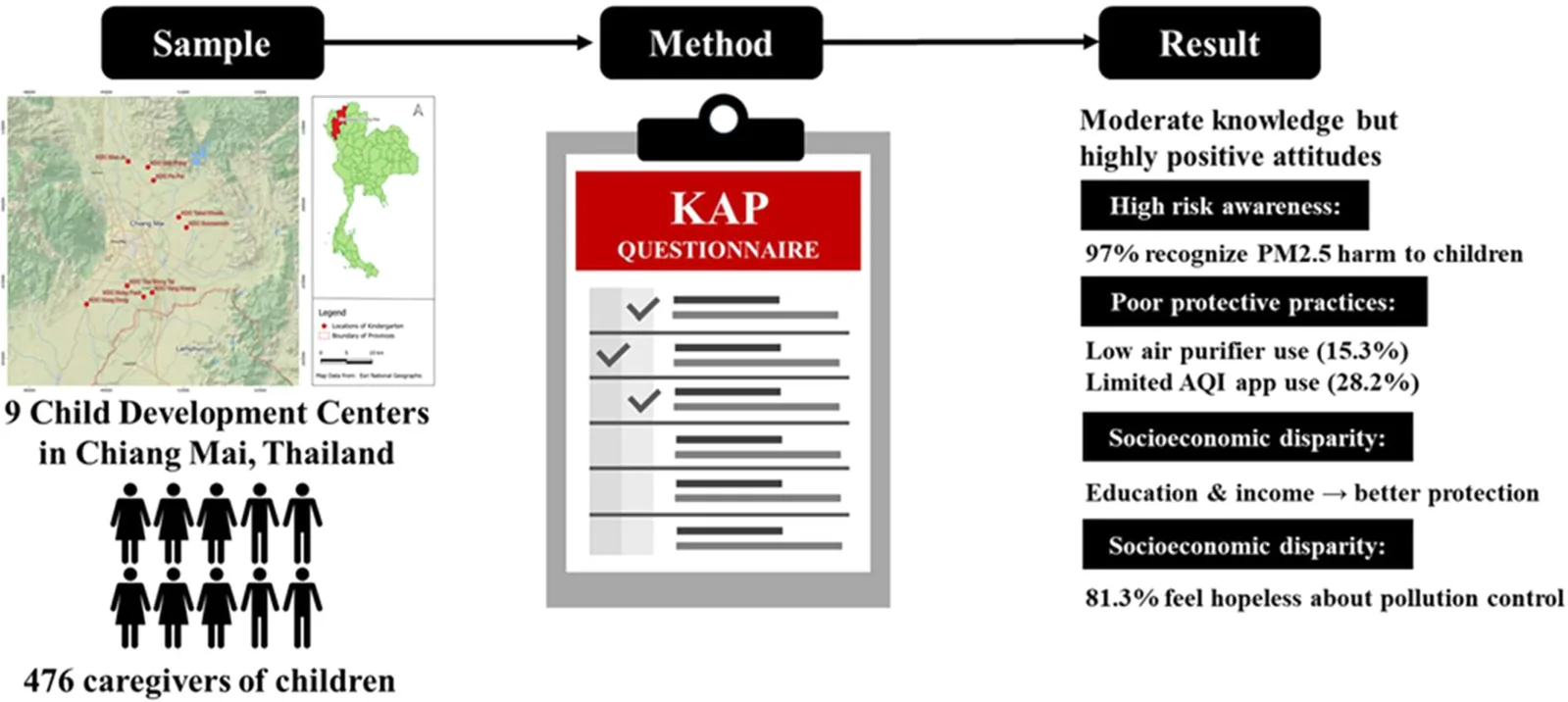

## 1 Introduction

Air pollution has emerged as one of the most pressing environmental health challenges globally, with fine particulate matter (PM_2.5_) recognized as a significant contributor to morbidity and mortality, particularly in developing regions [1]. PM_2.5_, comprising airborne particles with diameters <2.5 µm, can penetrate deep into the lungs and enter the bloodstream, posing serious health risks to vulnerable populations, especially young children [[2], [3], [4], [5], [6], [7]]. Beyond air pollution, children’s health vulnerabilities are compounded by climate change; higher and more frequent environmental hazards such as heatwaves, droughts, and extreme weather events can exacerbate respiratory and other health problems, to which children are particularly vulnerable because their respiratory and immune systems are still developing, making them less able to cope with environmental stressors [8,9].

Northern Thailand is a region acutely affected by seasonal air pollution, primarily driven by agricultural burning, forest fires, and transboundary haze [10]. The phenomenon of "smoke haze" typically peaks between January and April, with PM_2.5_ levels frequently exceeding national and international safety thresholds [11,12]. Children in this region are exposed repeatedly to high concentrations of PM_2.5_, placing a significant burden on families and local healthcare systems [13,14]. Additionally, climate change could increase the frequency of such pollution episodes and other environmental stressors, worsening risks to children's health in this sensitive region [8].

Demographics of Northern Thailand include dominant Northern Thai communities, various hill tribes (including the Hmong, Karen, Lahu, and Akha), and many Shan migrants from Myanmar [15,16]. However, despite pollution harm reduction efforts, awareness and protective behaviors differ across these groups, likely influenced by cultural, socioeconomic, and educational factors [17]. Therefore, this study focuses on caregivers of young children in rural and peri‑urban settings within these communities to measure PM_2.5_-related awareness and protective behaviors. Therefore, this study aimed to assess PM2.5-related knowledge, attitudes, and practices among caregivers of young children in rural and peri‑urban communities of Northern Thailand and to examine socio-demographic factors associated with these outcomes. By conducting the investigation in rural and peri‑urban settings, the study can consider diverse social, cultural, and environmental aspects that are less visible in Thai studies, which are currently carried out mainly on urban populations. In exploring these under-researched communities, this research adds detail to observed differences in environmental health literacy about PM2.5, building upon and enriching prior KAP research.

## 2 Methods

### 2.1 Study setting, population, study tool, and data collection method

**Fig. 1:**
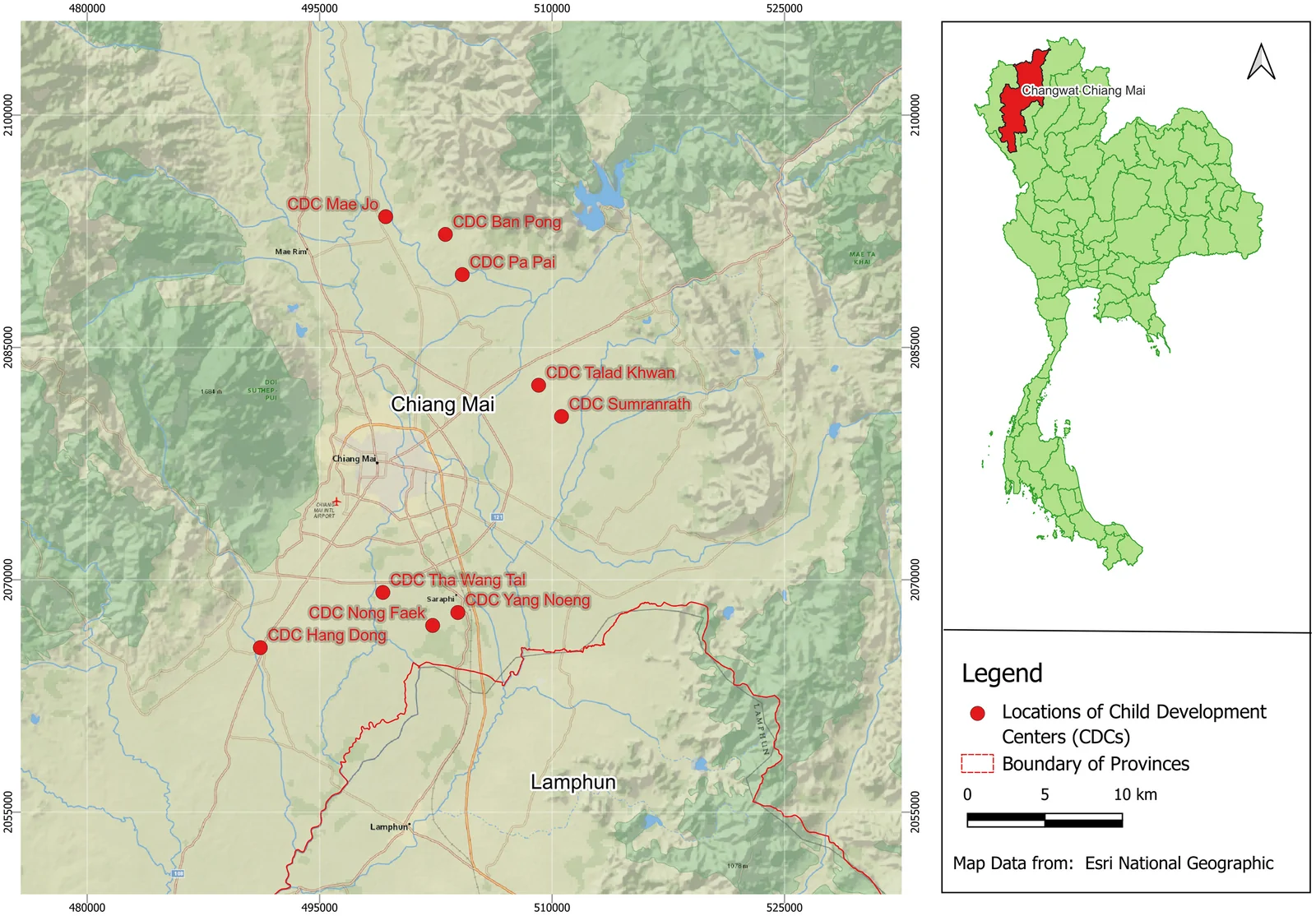
Map of the study locations.

This study used a cross-sectional design to assess environmental health literacy, protective behaviors, and the acceptability of government-led adaptation measures for PM_2.5_ exposure. The study was conducted in peri‑urban and rural districts of Chiang Mai Province (map in Fig. 1), Northern Thailand, a region severely affected by seasonal haze from wildfires and agricultural burning.

A total of nine child development centers were purposively selected for this study based on their geographic locations and our prior research experience. These centers are situated in rural areas predominantly engaged in agricultural activities and in peri‑urban areas undergoing new housing developments and industrial expansion, enabling us to capture a diverse range of environmental and socio-economic contexts. Additionally, our familiarity with the local authorities and community members from previous research facilitated effective coordination and engagement, ensuring smooth data collection and collaboration throughout the study.

The target population consisted of primary caregivers (parents or grandparents) of children aged 2–5 years enrolled at nine Child Development Centers (CDCs). The sample size for the Knowledge, Attitude, and Practice (KAP) survey was determined as 383 using the formula n = Z^2^ P(1-P) / d^2^, assuming a 95% confidence level (Z = 1.96), a precision error (d) of 0.05 [18], and an estimated good knowledge proportion (P) of 0.53 based on previous research in Northern Thailand [19]. Accounting for a 15% non-response rate, the minimal recruitment target was 440 participants. A consecutive convenience sampling approach was used, whereby all eligible caregivers present during recruitment periods at the selected CDCs were invited to participate. Since recruitment was conducted throughout the study period, all eligible and consenting caregivers encountered at the selected CDCs were included rather than enrollment being terminated once the minimum target was reached. The final sample size was 476. Caregivers were recruited at Child Development Centers (CDCs) during child drop-off and pick-up times. Subsequently, the study team scheduled appointments to conduct questionnaire interviews either at the participants’ homes or at the CDCs. Translators were engaged to assist caregivers who were not proficient in Thai during the questionnaire interviews. A structured 46-item Knowledge, Attitude, and Practice (KAP) questionnaire was adapted from previously validated instruments [[18], [19], [20], [21]] and pretested with a separate sample of caregivers (N = 30) who were excluded from the main survey. The pretest demonstrated high internal consistency, with Cronbach’s alpha coefficients of 0.868, 0.878, and 0.889 for the knowledge, attitude, and practice domains, respectively. The final version of the questionnaire was translated from English to Thai and then back-translated to English to ensure accuracy. The final survey instrument consisted of four sections: (1) socio-demographic characteristics (11 items), (2) knowledge regarding health impacts and air quality monitoring (13 items), (3) attitudes toward risk perception and trust in government (10 items), and (4) preventive practices and activity modification (12 items). Data collection was conducted through face-to-face interviews between September and October 2025.

Scoring procedures for the KAP domains were predefined. The knowledge domain consisted of 13 items, including one multiple-response question assessing awareness of population groups at risk of haze-related illness. For this question, one point was awarded for each correctly identified at-risk group. Because “everyone” was considered the most comprehensive correct response, respondents selecting this option were awarded five points to reflect recognition that PM_2.5_ exposure can affect the entire population. The maximum attainable knowledge score was 22 points, with higher scores indicating greater knowledge regarding PM_2.5_-related health risks and preventive measures. The attitude domain consisted of 10 statements scored on a four-point Likert scale ranging from 1 (strongly disagree) to 4 (strongly agree), yielding a total score range of 10–40. Negatively worded attitude items were reverse-coded before score calculation so that higher scores consistently reflected more positive attitudes toward PM_2.5_ prevention and protection. The practice domain consisted of eight preventive behavior items scored on a four-point frequency scale, yielding a maximum possible score of 32. Negatively worded practice items were reverse-coded where appropriate so that higher scores indicated more protective practices. Conditional questions related to air purifier ownership and filter replacement were collected for descriptive purposes only and were not included in the calculation of practice scores.

### 2.2 Data analysis

All statistical analyses were conducted using RStudio (version 2025.09.0 + 387; R Foundation for Statistical Computing, Vienna, Austria). Data were screened for missing values, outliers, and coding inconsistencies prior to analysis. No missing values were identified for the variables included in the analyses. Consequently, all 476 participants were retained in the descriptive and multivariable analyses, and no imputation procedures were required.

Descriptive statistics were used to summarize participants’ sociodemographic characteristics and children’s health-related symptoms. Continuous variables were expressed as mean and standard deviation (SD), while categorical variables were summarized as frequencies and percentages. To illustrate the distribution of KAP scores, raincloud-style plots combining violin, box, and jitter elements were generated to visualize score distributions across domains. Mean KAP scores were also summarized across participant subgroups (e.g., sex, education level, income, ethnicity, and residential area) to describe variations in KAP patterns. Correlations between KAP domains were assessed using Pearson correlation coefficients and visualized using a correlation matrix heatmap.

KAP scores were derived by summing responses across multiple questionnaire items and were analyzed as approximately continuous dependent variables. Given the relatively wide score ranges and the large sample size, multiple linear regression was considered an appropriate and interpretable approach for examining factors associated with knowledge, attitude, and practice scores. Multiple linear regression models were constructed separately for Knowledge, Attitude, and Practice scores to identify associated factors. Independent variables included sex, age, nationality, ethnicity, education level, household income, residential area (peri‑urban or rural), duration of residence in the community (since birth, 1–5 years, 6–10 years, or >10 years), number of preschool-aged children, and the presence of PM_2.5_-related health events (with at least one of these clinical conditions: irritation of the eyes; irritation of the nose, runny nose; irritation of the throat, coughing, and difficulty breathing; asthma exacerbation; increased respiratory infections, such as pneumonia; developmental delays; attention disorders; and other cognitive impacts; fatigue and headaches; aggravation of allergies or rash; and worsening of heart disease). Categorical predictors were dummy-coded with clearly defined reference categories.

Regression assumptions were evaluated through visual inspection of residual-versus-fitted plots and normal probability plots. These diagnostic assessments indicated no major departures from linearity or normality assumptions, although mild heteroskedasticity was observed in some models. Multicollinearity was assessed using variance inflation factors (VIF), with values <5 indicating acceptable levels. To account for the observed minor heteroskedasticity and improve the robustness of statistical inference, heteroskedasticity-consistent (HC3) robust standard errors were applied. Model performance was evaluated using the F-statistic, coefficient of determination (R^2^ and adjusted R^2^), residual standard error (RSE), and Akaike Information Criterion (AIC). Regression results were reported as unstandardized beta coefficients (β) with 95% confidence intervals (CI) and *p*-values. A *p*-value <0.05 was considered statistically significant.

The study was approved by the Ethics Committee of the Faculty of Tropical Medicine (MUTM: 2025–030–01). All participants provided informed consent, were assured of their right to withdraw without consequence, and were guaranteed data confidentiality and privacy in accordance with international ethical standards.

## 3 Results

### 3.1 Sociodemographic profile of participants

Of the 476 caregivers surveyed, most were female (63.9%), with a mean age of 35.1 ± 8.1 years (range: 18–69). The majority were Thai nationals (68.5%), while 31.5% were non-Thai, primarily Burmese (16.6%) or hill-tribe (7.6%) in ethnicity. About one-quarter (25.4%) had no formal education, and 23.1% held a diploma or higher qualification. Regarding household income, 41.2% reported a slightly low monthly income (5001–10,000 THB). Residency was predominantly rural (59.7%), while 31.5% had moved into their current community within the past five years.

Households had an average of 1.2 ± 0.4 preschool-aged children. During haze episodes, nasal irritation or runny nose (86.1%) was the most commonly reported symptom among children, followed by eye irritation (60.7%) and throat irritation or cough (59.0%). Less frequent but notable manifestations included flare-ups of chronic allergic conditions or asthma (18.7%), respiratory infections such as pneumonia (6.5%), and fatigue or headache (6.9%) (Table 1).

**Table 1:** Sociodemographic characteristics of caregivers of children attending child development centers (n = 476).

Variable	n (%)/ Mean ± SD (Range)
**Sex**	
Male	172 (36.1)
Female	304 (63.9)
**Age (Years)**	
Mean ± SD (Range)	35.1 ± 8.1 (18–69)
**Nationality**	
Thai	326 (68.5)
Non-Thai	150 (31.5)
**Ethnicity**	
Thai	305 (64.1)
Hill tribe	36 (7.6)
Burmese	79 (16.6)
Other	56 (11.8)
**Educational level**	
No formal education	121 (25.4)
Primary school	66 (13.9)
Secondary school	86 (18.1)
High school	93 (19.5)
Diploma and above	110 (23.1)
**Monthly household income** (**THB**#)	
Low (<5000 THB)	29 (6.1)
Slightly low (5001 – 10,000 THB)	196 (41.2)
Average (10,001 – 15,000 THB)	164 (34.5)
High (>15,000 THB)	87 (18.3)
**Residency location**	
Peri-urban	192 (40.3)
Rural	284 (59.7)
**Duration of residence**	
Since birth	137 (28.8)
Moved in ≤ 5 years	150 (31.5)
Moved in 6–10 years	95 (20.0)
Moved in > 10 years	94 (19.8)
**Number of preschool-aged children in the household**	
Mean ± SD (Range)	1.2 ± 0.4 (1–5)
Children’s symptoms during haze episodes*	
Eye irritation	289 (60.7)
Nasal irritation or runny nose	410 (86.1)
Throat irritation, cough, or breathing difficulty	281 (59.0)
Exacerbation of chronic disease (e.g., allergy, asthma)	89 (18.7)
Fatigue or headache	33 (6.9)
Respiratory infection (e.g., pneumonia)	31 (6.5)
Developmental or psychological problems	18 (3.8)
Heart-related symptoms	17 (3.6)

* Multiple responses allowed.

# 1 USD = 32 THB.

### 3.2 Knowledge about haze and PM_2.5_

In the domain of knowledge about haze and PM_2.5_, 94.3% of caregivers correctly recognized effects on children’s respiratory systems, and 90.8% acknowledged increased vulnerability of children with heart or lung disease. Correct responses related to protective measures and indoor air control were lower: 41.8% identified that regular face masks are insufficient against fine dust; 25.8% knew not all indoor plants absorb haze particles; 9.7% recognized that an orange AQI level indicates elevated pollution without mandating cessation of outdoor activities; 39.3% identified the PM_2.5_ standard threshold; and 46.6% knew about metal components in haze. Identification of at-risk groups varied: children (58.0%), everyone (56.5%), older adults (42.9%), people with respiratory diseases (31.1%), and people with heart diseases (16.8%) (Table 2).

**Table 2:** Knowledge about haze and PM_2.5_ among caregivers (n = 476).

Knowledge statement	n (%) of correct responses
In general, which groups are at risk of illness caused by haze? a	
Children	276 (58.0)
Older adults	204 (42.9)
People with respiratory diseases	148 (31.1)
People with heart diseases	80 (16.8)
People with mental health problems	52 (10.9)
Everyone	269 (56.5)
Haze affects children’s respiratory systems and can penetrate deep into the lungs.	449 (94.3)
Haze can cause stress and anxiety in children.	310 (65.1)
Children’s learning ability declines during periods of high haze.	276 (58.0)
The PM_2.5_ level of 50 µg/m³ is the 24-hour average standard.	187 (39.3)
Wearing a regular face mask is sufficient to protect against fine dust particles. b	199 (41.8)
All indoor plants can absorb haze particles. b	123 (25.8)
An AQI in orange means outdoor activities are prohibited. b	46 (9.7)
Air-conditioned rooms can prevent dust particles of all sizes. b	192 (40.3)
If a room has no air conditioner, the fan should be used and windows should be opened. b	144 (30.3)
Any air purifier can be used regardless of room size or condition. b	285 (59.9)
Children with heart and/or lung disease are more affected by haze.	432 (90.8)
Haze contains heavy metals such as lead, cadmium, chromium, and nickel.	222 (46.6)

a Multiple responses allowed.

b Negatively worded items (reverse-scored).

### 3.3 Attitudes toward PM_2.5_ pollution and preventive measures

Attitudinal responses indicated that 95.8% of caregivers agreed that monitoring PM2.5 levels is important; 97.0% recognized PM2.5 as a health risk to children. Concerns about impacts on children’s lungs and hearts were reported by 95.8%, and 95.3% noted worry when the sky appeared gray from haze. Concern for personal and family health during prolonged periods of high PM2.5 was expressed by 95.1%. Regarding preventive beliefs, 87.3% disagreed that staying indoors with an air purifier alone is sufficient protection, while 81.3% reported feelings of hopelessness during high PM2.5 levels. Additionally, 89.0% believed municipal policies could effectively address PM2.5 pollution (Table 3).

**Table 3:** Attitudes toward PM_2.5_ and haze pollution among caregivers (n = 476).

Attitude statement	Strongly disagree	Disagree	Agree	Strongly agree
	n (%)	n (%)	n (%)	n (%)
Checking PM_2.5_ levels in one’s residential area is important.	14 (2.9)	6 (1.3)	270 (56.7)	186 (39.1)
Children under your care should wear masks to protect against PM_2.5_ while at the child development center.	15 (3.2)	94 (19.7)	299 (62.8)	68 (14.3)
It is sufficient for children to stay inside a child development center building equipped with an air purifier to be protected from PM_2.5_. *	104 (21.8)	312 (65.5)	49 (10.3)	11 (2.3)
You think that PM_2.5_ poses a health risk to your child(ren) under your care.	8 (1.7)	7 (1.5)	253 (53.2)	208 (43.7)
You are concerned about the effects of PM_2.5_ on children’s lungs and hearts.	11 (2.3)	9 (1.9)	252 (52.9)	204 (42.9)
You are satisfied with your province’s PM_2.5_ prevention measures.	26 (5.5)	82 (17.2)	300 (63.0)	68 (14.3)
You feel worried about your child when the sky turns gray due to haze.	12 (2.5)	8 (1.7)	240 (50.4)	216 (45.4)
You are concerned about your and your family’s health when PM_2.5_ levels are high for consecutive days.	14 (2.9)	9 (1.9)	202 (42.4)	251 (52.7)
You feel hopeless when PM_2.5_ levels are high because you believe you cannot adequately protect your child. *	16 (3.4)	73 (15.3)	264 (55.5)	123 (25.8)
You believe municipal policies can effectively solve the PM2.5 problem.	13 (2.7)	39 (8.2)	330 (69.3)	94 (19.7)

* Negatively worded items (reverse-scored).

### 3.4 Behaviors and preventive practices toward PM_2.5_ exposure

With respect to monitoring PM_2.5_ levels, most caregivers reported limited engagement with air-quality information tools and prevention technologies. More than two-thirds (71.8%) did not use any application or website to check PM2.5 levels, and only a small proportion used Air4Thai (9.2%) or IQAir (9.0%).

Air purifier ownership was reported by 15.3% of caregivers, with 57.5% of those replacing filters within the past year. Preventive practices varied: 86.1% followed health information from authorities, 94.1% increased indoor ventilation when air quality improved, and 73.7% never kept doors and windows closed during haze periods (Table 4A, Table 4B). Protective behaviors included limiting children’s outdoor activities on high PM2.5 days (97.9%) and having children wear protective masks outdoors (77.3%).

**Table 4A:** Device ownership and information-checking behavior (n = 476).

Variable	n (%)/ Mean ± SD (Range)
**Use of applications or websites to check PM_2.5_** *	
None	342 (71.8)
Air4Thai	44 (9.2)
IQAir	43 (9.0)
Northern Thailand Air Quality Health Index	8 (1.7)
Other	39 (8.2)
**Ownership of air purifiers**	
None	403 (84.7)
Yes (Range)	73 (15.3) (1–4)
**Filter replacement among air purifier owners** (n = 73)	
Never replaced	20 (27.4)
Replaced within the past year	42 (57.5)
Replaced more than one year ago	11 (15.1)

* Multiple responses allowed. Percentages for filter replacement were calculated among caregivers who reported owning at least one air purifier (n = 73).

**Table 4B:** Frequency of preventive behaviors against air pollution (n = 476).

Behavior	Never	Rarely	Sometimes	Always
	n (%)	n (%)	n (%)	n (%)
Check PM_2.5_ levels through mobile applications or websites	186 (39.1)	26 (5.5)	188 (39.5)	76 (16.0)
Limit children’s outdoor activities on high PM_2.5_ days	5 (1.1)	4 (0.8)	218 (45.8)	249 (52.3)
Keep windows and doors closed during haze periods	351 (73.7)	30 (6.3)	84 (17.6)	11 (2.3)
Have children wear protective masks when outdoors	63 (13.2)	45 (9.5)	272 (57.1)	96 (20.2)
Follow health or environmental information from authorities	28 (5.9)	38 (8.0)	197 (41.4)	213 (44.7)
Participate in community or school campaigns about air pollution prevention	41 (8.6)	32 (6.7)	234 (49.2)	169 (35.5)
Increase ventilation and indoor air circulation when air quality improves	9 (1.9)	19 (4.0)	238 (50.0)	210 (44.1)
Educate children about air pollution and self-protection	144 (30.3)	63 (13.2)	213 (44.7)	56 (11.8)

### 3.5 Distribution of knowledge, attitude, and practice scores

**Fig. 2:**
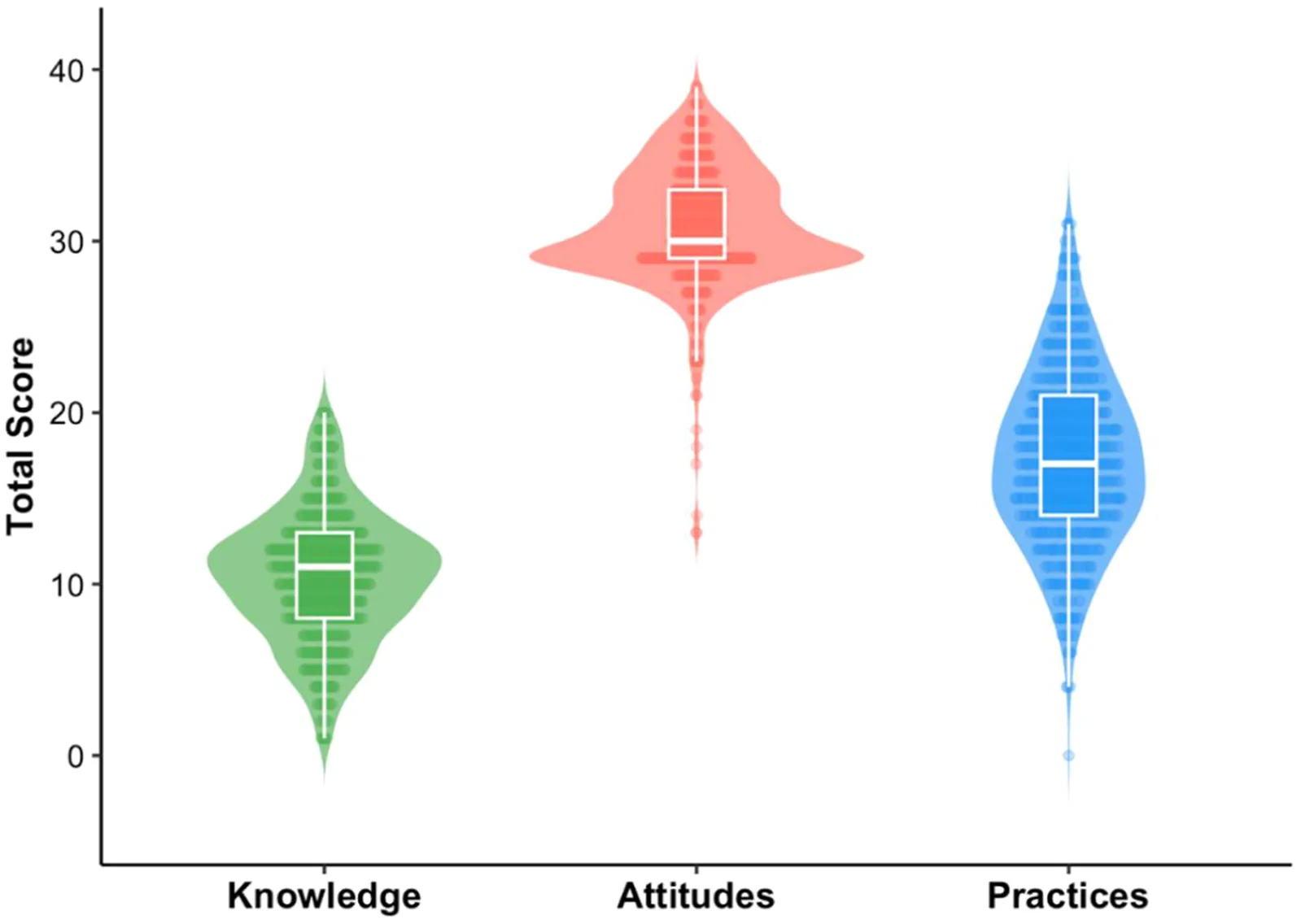
Distribution of knowledge, attitude, and practice scores among caregivers of preschool children. Note: The raincloud plots illustrate the distributions, central tendencies, and variability of the KAP scores. Because each KAP domain has a different maximum score, absolute values should not be directly across domains. The plots are intended to visualize the distribution and spread of scores within each domain.

Knowledge scores ranged from 1 to 20 out of 22, with a mean of 10.44 ± 3.80 and a median of 11. Attitude scores ranged from 13 to 39 out of 40, with a mean of 30.74 ± 3.57 and a median of 30. Practice scores ranged from 0 to 31 out of 32, with a mean of 17.33 ± 5.22 and a median of 17. The distributions of the three domains are presented in Fig. 2.

### 3.6 Correlation between knowledge, attitude, and practice scores

**Fig. 3:**
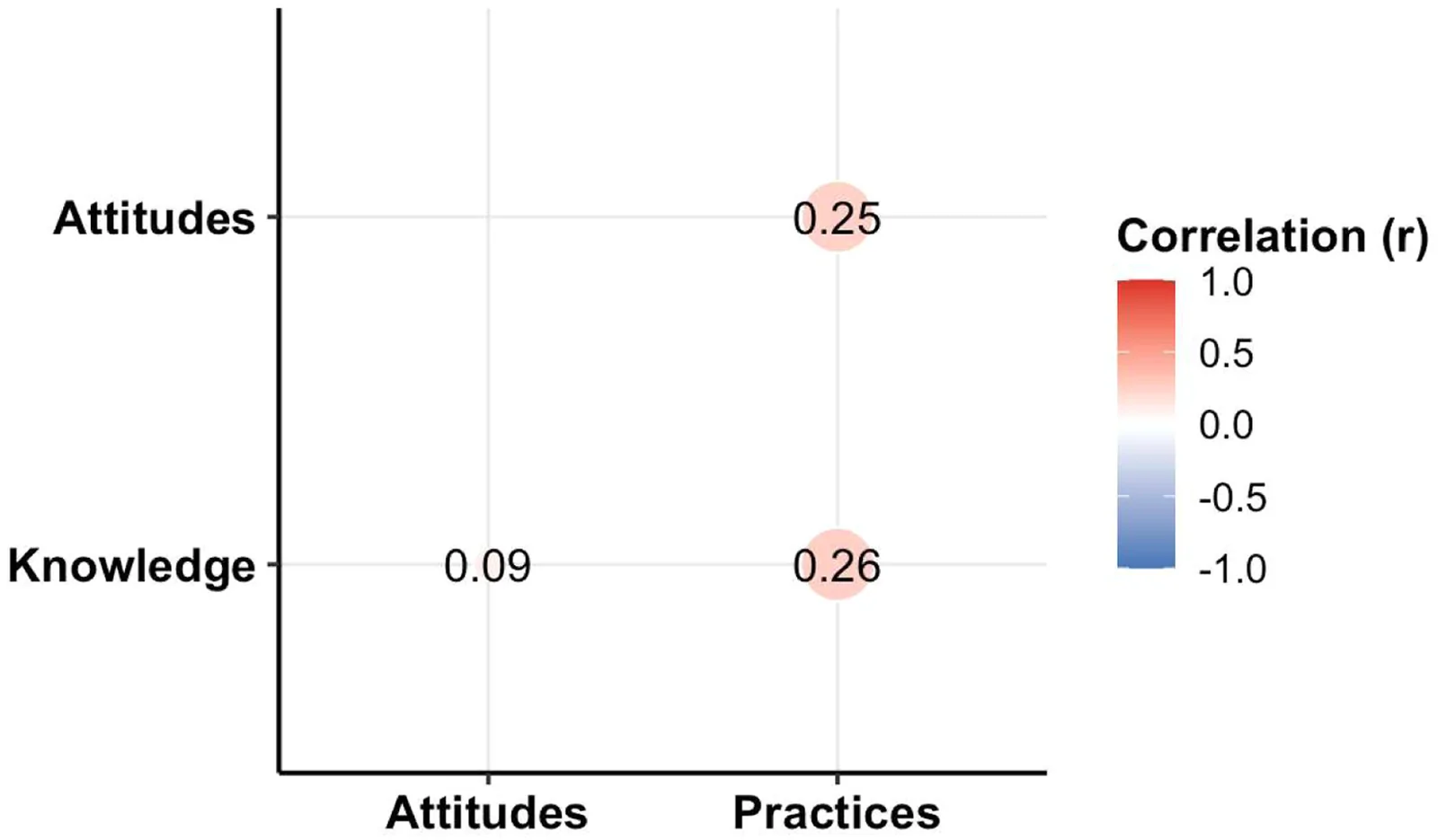
Correlation between knowledge, attitude, and practice scores. Note: Pearson correlation coefficients (r) are displayed. Circle size reflects the strength of correlation, and color indicates direction (red = positive; blue = negative).

Pearson correlation coefficients were r = 0.26 between knowledge and practice scores, 0.09 between knowledge and attitude scores, and a weak 0.25 between attitudes and practice scores (r = 0.25) (Fig. 3).

### 3.7 Knowledge, attitude, and practice scores by participant characteristics

Mean KAP scores differed by participant characteristics (Table 5). Female caregivers had slightly higher knowledge (10.55 ± 3.75) and practice (17.63 ± 5.42) scores than males (knowledge 10.26 ± 3.89; practice 16.80 ± 4.83). Thai nationals had higher knowledge (10.92 ± 3.72) and practice scores (18.45 ± 5.20) than non-Thai caregivers (knowledge: 9.40 ± 3.77; practice: 14.89 ± 4.39). Knowledge and practice scores differed across educational categories, with the highest mean scores observed among caregivers with a diploma or higher qualification (knowledge 11.45 ± 3.59; practice 20.74 ± 4.65). Differences in knowledge and practice scores were also observed across household income categories, with the highest mean scores reported among caregivers in the highest income group. Attitude scores were generally consistent across groups, with slightly higher attitudes among rural residents (31.00 ± 3.69) than among peri‑urban residents (30.37 ± 3.37). Caregivers reporting PM2.5-related child health symptoms had marginally higher knowledge scores (10.47 ± 3.76) compared to those without (9.00 ± 5.33).

**Table 5:** Mean knowledge, attitude, and practice scores by participant characteristics (n = 476).

Characteristic	Knowledge	Attitudes	Practices
	Mean ± SD	Mean ± SD	Mean ± SD
**Sex**			
Male	10.26 ± 3.89	30.84 ± 3.51	16.80 ± 4.83
Female	10.55 ± 3.75	30.69 ± 3.61	17.63 ± 5.42
**Nationality**			
Thai	10.92 ± 3.72	30.74 ± 3.61	18.45 ± 5.20
Non-Thai	9.40 ± 3.77	30.75 ± 3.51	14.89 ± 4.39
**Ethnicity**			
Thai	11.03 ± 3.64	30.69 ± 3.64	18.73 ± 5.11
Hill tribe	8.83 ± 4.16	31.22 ± 3.67	15.06 ± 4.04
Burmese	9.25 ± 3.95	30.57 ± 3.47	14.27 ± 4.91
Other	9.95 ± 3.52	30.98 ± 3.31	15.46 ± 3.90
**Educational level**			
No formal education	9.50 ± 4.07	30.78 ± 3.51	14.58 ± 4.65
Primary school	9.55 ± 3.59	30.80 ± 3.78	15.85 ± 4.60
Secondary school	10.35 ± 3.53	30.15 ± 3.67	17.23 ± 4.80
High school	11.19 ± 3.67	30.60 ± 3.67	18.02 ± 4.98
Diploma and above	11.45 ± 3.59	31.25 ± 3.34	20.74 ± 4.65
**Monthly household income (THB)**			
Low (<5000 THB)	9.90 ± 3.13	29.62 ± 2.87	15.62 ± 5.50
Slightly low (5001 – 10,000 THB)	10.41 ± 3.83	30.60 ± 3.77	15.78 ± 4.86
Average (10,001 – 15,000 THB)	9.96 ± 3.90	30.80 ± 3.48	17.45 ± 4.69
High (>15,000 THB)	11.61 ± 3.55	31.32 ± 3.43	21.17 ± 4.91
**Residency location**			
Peri-urban	10.82 ± 3.73	30.37 ± 3.37	18.83 ± 4.99
Rural	10.19 ± 3.83	31.00 ± 3.69	16.31 ± 5.14
**Duration of residence**			
Since birth	10.47 ± 3.77	30.31 ± 3.84	18.11 ± 5.09
Moved in ≤ 5 years	10.35 ± 3.78	31.30 ± 3.60	17.39 ± 5.36
Moved in 6–10 years	10.78 ± 3.81	30.64 ± 3.67	17.63 ± 5.85
Moved in > 10 years	10.20 ± 3.91	30.60 ± 2.89	15.80 ± 4.19
**Presence of health-related events due to PM_2.5_**			
**No**	9.00 ± 5.33	32.10 ± 3.51	15.50 ± 4.81
**Yes**	10.47 ± 3.76	30.71 ± 3.57	17.37 ± 5.23

SD: standard deviation; Note: KAP scores are not directly comparable between domains due to differing maximum possible scores.

### 3.8 Factors associated with knowledge, attitudes, and practices toward PM**_2.5_**

Multivariate regression results indicated the knowledge model was statistically significant (adjusted R^2^ = 0.056) with education and income as significant predictors. Caregivers with primary education scored lower on knowledge than those with a diploma or higher (β = −1.51; 95% CI: −2.87 to −0.16; p = 0.029). Slightly low income (β = 1.05; 95% CI: 0.23 to 1.86; p = 0.012) and high income (β = 1.06; 95% CI: 0.02 to 2.10; p = 0.045) were associated with higher knowledge compared to average income. Age, sex, nationality, ethnicity, residence, duration of residence, number of children, and health events were not significant.

The attitude model was not statistically significant overall (adjusted R^2^ = 0.018). Coefficient estimates from the model indicated higher attitude scores among rural residents (β = 0.92; 95% CI: 0.16 to 1.68; p = 0.017) and participants who had resided in the community for five years or less (β = 0.89; 95% CI: 0.00 to 1.78; p = 0.049), whereas secondary education was associated with lower attitude scores compared with diploma or higher education (β = −1.29; 95% CI: −2.40 to −0.19; p = 0.022). No other variables showed statistically significant associations.

The practices model had an adjusted R^2^ = 0.217. Education and income significantly predicted practice scores. Compared to diploma or higher education, no formal education (β = −3.06; 95% CI: −4.99 to −1.13; p = 0.002), primary (β = −2.56; 95% CI: −4.26 to −0.86; p = 0.003), and secondary education (β = −1.96; 95% CI: −3.41 to −0.53; p = 0.008) were associated with lower practice scores. High income corresponded to higher practice scores (β = 2.35; 95% CI: 1.05 to 3.66; p < 0.001). The number of preschool-aged children in the household showed a positive but non-significant association with practice scores (β = 0.85; 95% CI: −0.11 to 1.82; p = 0.083). No significant effects were found for sex, age, residence, duration, or health events (Table 6).

**Table 6:** Multivariable linear regression analysis of factors associated with knowledge, attitudes, and practices toward PM_2.5_ among caregivers (n = 476).

Characteristic	Knowledge		Attitudes		Practices	
	β (95% CI)	*p*	β (95% CI)	*p*	β (95% CI)	*p*
**Age** (years)	−0.02 (−0.07, 0.02)	0.313	−0.00 (−0.05, 0.04)	0.887	−0.03 (−0.08, 0.03)	0.371
**Sex**						
Male	Reference		Reference		Reference	
Female	0.14 (−0.58, 0.86)	0.698	−0.04 (−0.73, 0.66)	0.919	0.62 (−0.29, 1.52)	0.180
**Nationality**						
Thai	0.49 (−1.43, 2.41)	0.614	0.98 (−0.86, 2.82)	0.296	−0.58 (−2.98, 1.82)	0.635
Non-Thai	Reference		Reference		Reference	
**Ethnicity**						
Thai	1.13 (−1.00, 3.25)	0.614	−0.74 (−2.78, 1.30)	0.474	2.49 (−0.18, 5.15)	0.067
Hill tribe	−0.53 (−2.15, 1.20)	0.524	0.61 (−0.94, 2.17)	0.439	0.10 (−1.93, 2.13)	0.922
Burmese	Reference		Reference		Reference	
Other	0.85 (−0.44, 2.14)	0.197	0.48 (−0.76, 1.72)	0.446	1.14 (−0.47, 2.75)	0.165
**Educational level**						
No formal education	−1.08 (−2.62, 0.46)	0.169	−0.94 (−2.42, 0.54)	0.211	**−3.06 (−4.99, −1.13)**	0.002*
Primary school	**−1.51 (−2.87, −0.16)**	0.029*	−0.62 (−1.92, 0.69)	0.352	**−2.56 (−4.26, −0.86)**	0.003*
Secondary school	−0.91 (−2.06, 0.25)	0.124	**−1.29 (−2.40, −0.19)**	0.022*	**−1.96 (−3.41, −0.53)**	0.008*
High school	−0.28 (−1.38, 0.81)	0.611	−0.68 (−1.73, 0.38)	0.208	**−1.56 (−2.93, −0.19)**	0.026*
Diploma and above	Reference		Reference		Reference	
**Monthly household income (THB)**						
Low	0.57 (−0.93, 2.07)	0.457	−1.25 (−2.68, 0.19)	0.089	−0.74 (−2.62, 1.13)	0.436
Slightly low	**1.05 (0.23, 1.86)**	0.012*	−0.34 (−1.12, 0.44)	0.396	−0.56 (−1.58, 0.46)	0.281
Average	Reference		Reference		Reference	
High	**1.06 (0.02, 2.10)**	0.045*	0.32 (−0.68, 1.31)	0.536	**2.35 (1.05, 3.66)**	<0.001*
**Residency location**						
Peri-urban	Reference		Reference		Reference	
Rural	0.10 (−0.69, 0.89)	0.803	**0.92 (0.16, 1.68)**	0.017*	−0.26 (−1.25, 0.73)	0.610
**Duration of residence**						
Since birth	Reference		Reference		Reference	
Moved in ≤ 5 years	0.30 (−0.63, 1.22)	0.527	**0.89 (0.00, 1.78)**	0.049*	0.16 (−1.00, 1.32)	0.782
Moved in 6–10 years	0.90 (−0.13, 1.92)	0.086	0.29 (−0.69, 1.27)	0.564	0.49 (−0.79, 1.77)	0.455
Moved in > 10 years	0.71 (−0.36, 1.78)	0.192	0.16 (−0.87, 1.18)	0.761	−0.49 (−1.82, 0.85)	0.476
**Number of preschool-aged children in the household**	−0.62 (−1.39, 0.15)	0.115	−0.57 (−1.31, 0.17)	0.131	0.85 (−0.11, 1.82)	0.083
**Presence of health-related events due to PM_2.5_**						
No	Reference		Reference		Reference	
Yes	1.17 (−1.26, 3.60)	0.344	−1.43 (−3.76, 0.90)	0.228	−0.61 (−3.65, 2.43)	0.694

*Significance at *p*-value <0.05.

## 4 Discussion

This study revealed a complex context of PM_2.5_ awareness among caregivers, characterized by great general concern but significant gaps in technical knowledge and practical application.

### 4.1 Awareness, knowledge gaps, and preventive practices

97 percent of caregivers reported that PM_2.5_ poses a health risk; however, some gaps emerged in technical knowledge of protection measures for individuals. For instance, false beliefs about the effectiveness of regular face masks or indoor plants in reducing PM_2.5_ exposure were observed. This is consistent with previous research that noted relatively high levels of awareness, but there was little technical data on pollution exposure and protective measures among other urban and peri‑urban Asian populations [[20], [21], [22]], Consistent with these findings, the great majority of caregivers were not users of air quality monitoring apps (71.8%), which likely indicated reliance on visual cues of haze exposure or on the sky's dark or bright color ranges. This behavior also confirms previous research findings that the likelihood of risk assessment is positively influenced when one experiences subjective perceptions of the environmental factors of concern, as objective exposure data sometimes are scarce [23].

### 4.2 Socioeconomic barriers and the practice trend

Results of the analyses confirmed correlations between socioeconomic characteristics – predominantly education and income – and self-reported participation in preventive activity. In line with the findings reported by Luangwilai et al. (2025) [24], low educational attainment was associated with lower practice scores. Ownership of air purifiers was low (15.3%), while only 9% reported regularly maintaining filters, suggesting potential economic constraints on improving indoor air quality. These findings align with Huang et al. (2024) [25] and Kausar et al. (2025) [26], who observed that implementing protective measures depends on economic and contextual factors, not limited to knowledge alone. Because of the cross-sectional design, the present results indicate associations rather than causation.

### 4.3 Psychological burden and the role of policy

A large proportion of the caregivers reported feeling a sense of hopelessness when PM_2.5_ levels were high, even when their attitudes towards protective measures were generally positive. Although these emotional responses were not quantitatively assessed with a well-validated psychological scale, this information suggests that some caregivers struggle to cope with environmental health risks. Previous publications (e.g., Bahrami et al., 2024 [27]) have connected perceived environmental stressors to psychological distress, which should be interpreted with caution given the proposed measures of this study. Importantly, a large majority of caregivers (89.0%) have confidence in municipal policies to combat air pollution. This may indicate that public demands for governmental action, along with private prevention endeavors, are also common, a pattern seen in other locations (e.g., India [28]).

### 4.4 Vulnerability and educational gaps

Lower knowledge, attitude, and practice (KAP) scores among non-Thai caregivers and those with lower educational attainment were found to reflect disparities in access to information and protective measures. Only 9.7% of caregivers correctly interpreted the orange AQI level, suggesting that current public health communication interventions were either less effective or insufficiently widespread. Similar concerns exist in other geographical contexts and with marginalized populations [29].

### 4.5 Study limitations

While the current study contributes important information on the knowledge, attitudes, and practices (KAP) of caregivers in Thailand regarding PM2.5 exposure, some limitations should be acknowledged. First, the cross-sectional nature of the study precludes causal inference; associations between the social determinants of health and KAP can be determined, but no temporal or causal relationships can be established. Second, preventive behavior data were self-reported and thus subject to social desirability bias, as participants may have overestimated their adherence to recommended health habits, such as mask-wearing and window-closing. Participants also may be affected by recall bias (i.e., they might not accurately remember or report their behaviors). It was also common for perceived behaviors not to correspond to real-time exposure. Third, the study population consisted of caregivers attending selected Child Development Centers (CDCs) in specific rural and peri‑urban catchment areas. This sampling strategy was restricted to caregiver drop-off and pick-up, which creates selection bias and ultimately limits generalizability beyond these geographic contexts and populations. There may be different KAP profiles depending on the topographies and regions of Thailand where biomass burning is predominant. Volunteer bias might also have influenced the outputs, as more health-conscious individuals are more likely to participate. Fourth, there may have been language and literacy challenges for respondents, especially non-Thai caregivers, that hindered their understanding of the survey instrument, which could ultimately affect the reliability of their responses. Although the questionnaire was pretested with a separate group, the construct validity of the adapted tool and the suitability of the scoring method for all subjects remain unknown. Overall, these limitations warrant caution when interpreting the findings and underscore the need for further longitudinal, methodologically robust studies to illuminate caregiver KAP regarding PM_2.5_ across different Thai contexts.

### 4.6 Policy implications and recommendations

These findings indicate that targeted measures are required to develop technical skills, thereby ensuring equitable access to good protective measures and supporting equity in protective knowledge. Policy implications for future development projects or interventions are warranted. Possible actions include government subsidies for HEPA-grade air purifiers and N95 masks for low-income households and schools. Developing simple, actionable AQI alerts in multiple languages might improve public awareness and increase willingness to act. In addition, policies that promote or require the use of high-efficiency air filtration systems in daycare sites or public buildings may help reduce indoor PM_2.5_ exposure among at-risk groups.

## 5 Conclusion

Caregiver knowledge, attitudes, and practices (KAP) assessment data revealed that knowledge levels were moderate, attitudes toward child protection were generally positive, and practices varied among participants. Practice scores were positively correlated with higher education level and income. They show associations of socio-economic status with caregiving without implying cause and effect. Future studies should consider tailoring interventions to the cultural and economic contexts of target populations to improve generalizability. Furthermore, integrating knowledge and practical resources may be critical to the design of public health interventions. These issues could be studied more in context-specific research in the future.

## Funding

This research has received funding support from the National Science, Research and Innovation Fund (NSRF) via the Program Management Unit for Human Resources & Institutional Development, Research and Innovation [B50G670097] to KT, the e-ASIA Joint Research Program [2026359], through funding provided by [National Health and Medical Research Council (NHMRC), Australia] to SV and Wellcome Trust (Grant Reference 310797/Z/24/Z) - Climate Attribution of Wildfire Smoke Impacts on Priority Population Health in Southeast Asia and Australia (CANBREATHE) to KT and SV.

## CRediT authorship contribution statement

**Suparat Phuanukoonnon:** Writing – review & editing, Writing – original draft, Validation, Investigation, Formal analysis, Data curation, Conceptualization. **Pyae Linn Aung:** Writing – review & editing, Validation, Methodology, Formal analysis. **Wissanupong Kliengchuay:** Writing – review & editing, Data curation, Conceptualization. **Sarima Niampardit:** Writing – review & editing, Visualization, Data curation. **Nuttapohn Kiangkoo:** Writing – review & editing, Data curation. **Rachaneekorn Mingkhwan:** Writing – review & editing, Data curation. **Paweena Aendo:** Writing – review & editing, Data curation. **Sawaeng Kawichai:** Writing – review & editing, Data curation. **Kotchakorn Khammoon:** Writing – review & editing, Data curation. **Tippawan Prapamontol:** Writing – review & editing, Supervision. **Nguyen Thi Kim Oanh:** Writing – review & editing, Supervision, Investigation. **Walaiporn Phonphan:** Writing – review & editing, Visualization. **Bin Jalaludin:** Writing – review & editing, Supervision, Investigation. **Sotiris Vardoulakis:** Writing – review & editing, Supervision, Investigation. **Kraichat Tantrakarnapa:** Writing – review & editing, Validation, Software, Project administration, Methodology, Funding acquisition, Conceptualization.

## Declaration of competing interest

The authors declare that they have no known competing financial interests or personal relationships that could have appeared to influence the work reported in this paper.
